# Identification of hub genes associated with spermatogenesis by bioinformatics analysis

**DOI:** 10.1038/s41598-023-45620-3

**Published:** 2023-10-27

**Authors:** Shuang Liu, Yan-chao Bian, Wan-lun Wang, Tong-Jia Liu, Ting Zhang, Yue Chang, Rui Xiao, Chuan-ling Zhang

**Affiliations:** 1 Inner Mongolia Key Laboratory of Molecular Pathology, Inner Mongolia Medical University, Huhhot, 010059 Inner Mongolia Autonomous Region China; 2 Department of Pharmacy, Inner Mongolia Medical University, Huhhot, 010110 Inner Mongolia Autonomous Region China

**Keywords:** Cancer genetics, Gene expression, Genetic markers

## Abstract

Spermatogenesis is a complex process related to male infertility. Till now, the critical genes and specific mechanisms have not been elucidated clearly. Our objective was to determine the hub genes that play a crucial role in spermatogenesis by analyzing the differentially expressed genes (DEGs) present in non-obstructive azoospermia (NOA) compared to OA and normal samples using bioinformatics analysis. Four datasets, namely GSE45885, GSE45887, GSE9210 and GSE145467 were used. Functional enrichment analyses were performed on the DEGs. Hub genes were identified based on protein–protein interactions between DEGs. The expression of the hub genes was further examined in the testicular germ cell tumors from the TCGA by the GEPIA and validated by qRT-PCR in the testes of lipopolysaccharide-induced acute orchitis mice with impaired spermatogenesis. A total of 203 DEGs including 34 up-regulated and 169 down-regulated were identified. Functional enrichment analysis showed DEGs were mainly involved in microtubule motility, the process of cell growth and protein transport. *PRM2, TEKT2, FSCN3, UBQLN3, SPATS1* and *GTSF1L* were identified and validated as hub genes for spermatogenesis. Three of them (*PRM2, FSCN3* and *TEKT2*) were significantly down-regulated in the testicular germ cell tumors and their methylation levels were associated with the pathogenesis. In summary, the hub genes identified may be related to spermatogenesis and may act as potential therapeutic targets for NOA and testicular germ cell tumors.

## Introduction

Infertility is a severe condition for the male or female reproductive system, affecting 10–15% of couples worldwide and about 50% of infertility cases are related to men^1–3^. More than 30 million men suffer from infertility in the world. However, the molecular mechanisms underlying male infertility have not been fully elucidated. It has been reported that many factors are associated with male infertility, including various congenital and acquired genitourinary disorders, hormone secretion disorders, multiple reproductive tract infections, genetic mutations, and excessive scrotal hyper-temperature^4–6^.

Spermatogenesis is a complex biological process involving mitotic cell division, meiosis cell division, and spermiogenesis^7^. Any morphological and pathological defects in the process of spermatogenesis can cause male infertility, such as a decline in sperm counts, malformation of sperm, and poor sperm motility. As reported, over 2000 genes are expressed primarily in male germ cells of mice^8^, and more than 400 of them including calcium binding tyrosine phosphorylation regulated (*Cabyr),* protein phosphatase 3, regulatory subunit B, alpha isoform (calcineurin B, type II) *(Ppp3r2),* DEAD box helicase 5*(Ddx5),* coiled-coil domain containing 63 *(Ccdc63),* chromodomain protein, Y chromosome-like (*Cdyl*) and Sad1 and UNC84 domain containing 3* (Sun3)* have been experimentally proven to be related to spermatogenesis and male infertility^9–14^. Current research on male infertility has focused on screening for DEGs^15,16^. The wide application of high-throughput sequencing technologies such as RNA sequencing has provided highly comprehensive and accurate results of DEGs in spermatogenesis using bioinformatics analysis. Therefore, finding key genes that affect male fertility will provide a theoretical basis for further treatment.

In the present study, we used a comprehensive bioinformatics method to screen key genes for male fertility, and their expression was experimentally verified. The results will provide new insight into the mechanisms of male fertility and enable us to find potential therapeutical targets for male infertility diseases such as oligospermia/azoospermia and testicular cancers.

## Result

### Identification of DEGs

**Figure 1:**
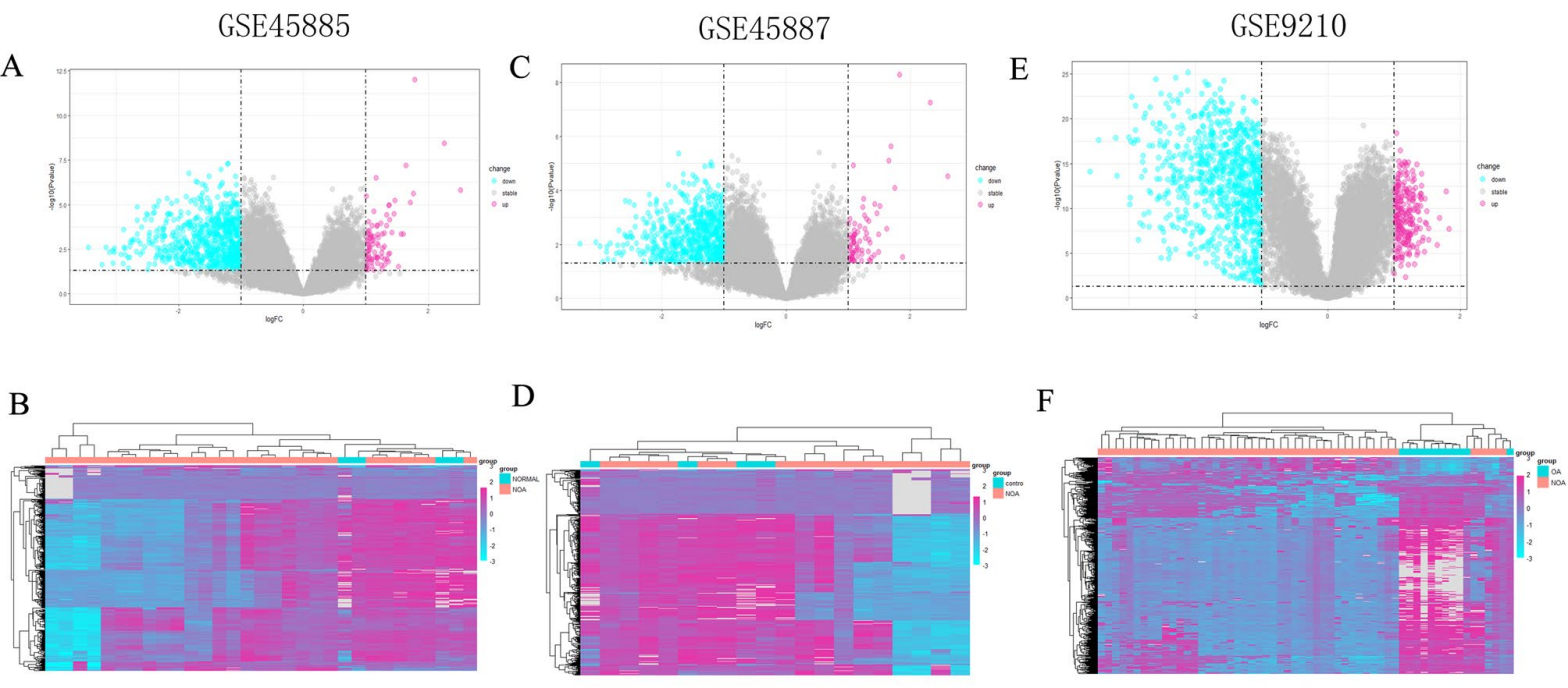
Volcano plots and heatmaps of DEGs in GSE45585, GSE45587 and GSE9210 datasets. Volcano plots of significant DEGs with |log_2_FC|> 1 in GSE45585 (**A**), GSE45587 (**B**) and GSE9210 (**C**) (*P* < 0.05). Blue: down-regulated DEGs; Purple: up-regulated DEGs; Grey: not significantly changed DEGs. Heatmaps of DEGs in GSE45585 (**D**), GSE45587 (**E**) and GSE9210 (**F**) datasets. Pink: NOA group; Blue: control group.

Firstly, the individual batch effects on datasets GSE45885, GSE45887 and GSE9210 were corrected and normalized (Supplementary Fig. 1A–C). Principal component analysis (PCA) was then performed to reduce the data dimensions for gene expression analysis (Supplementary Fig. 1D–F). Three microarray expression datasets include 90 NOA, 11 obstructive azoospermia (OA) and 8 normal samples (Table 1). In our analysis, 791 DEGs (86 up-regulated and 705 down-regulated genes) were identified in the GSE45585 dataset (Fig. 1A,B). In the GSE45587 dataset, a total of 723 DEGs with 76 up-regulated and 647 down-regulated genes were identified (Fig. 1C,D). A total of 1087 DEGs were identified in the GSE9210 dataset, of which 223 are up-regulated and 864 are down-regulated (Fig. 1E,F). Thus, we analyzed three datasets and identified 203 DEGs including 34 up-regulated and 169 down-regulated genes in NOA compared to OA and normal samples exhibiting normal spermatogenesis (|log_2_Fold change (FC)|> 1.0, *P adjusted(adj*) < 0.05). Besides, four datasets including the above three screening and one validation (GSE145467) datasets were normalized and together used for DEGs screening from NOA compared to OA and normal samples. Finally, 182 DEGs were screened (Supplementary Fig. 2). Interestingly, all of them were contained in the DEGs identified from three datasets analysis, which means that our results based on either three or four datasets are consistent and indicate that these identified DEGs may play key functions in NOA.

**Table 1:** Downloaded data from the GEO platform.

GSE profile	GPL platform	Organism	Sample source	Experiment Type	NOA case	Control case
GSE45885	GPL6244	Homo sapiens	testicular biopsy	Expression profiling by array	27	4(normal)
GSE45887	GPL6244	Homo sapiens	testicular biopsy	Expression profiling by array	16	4(normal)
GSE9210	GPL887	Homo sapiens	testicular biopsy	Expression profiling by array	47	11(OA)
GSE145467	GPL4133	Homo sapiens	testicular biopsy	Expression profiling by array	10	10(OA)

### Gene Ontology (GO) and functional enrichment analysis

**Figure 2:**
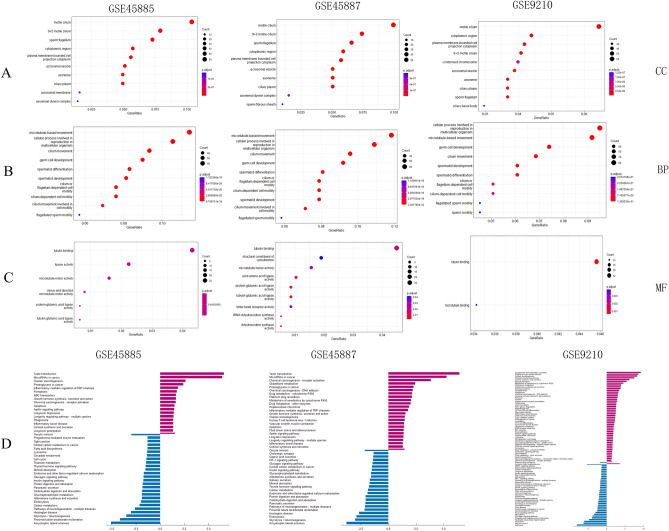
GO and KEGG enrichment analysis. GO term “cellular component (CC)” (**A**), GO term “biological process (BP)” (**B**) and GO term “molecular function (MF)”(**C**) for DEGs in GSE45585, GSE45587 and GSE9210 datasets. The size of the circle indicates the number of genes enriched in each GO term pathway. The red colour represents a lower *P* value. Blue represents higher. KEGG pathways enriched in GSE45585, GSE45587 and GSE9210 datasets (**D**). Blue: down-regulated DEGs enriched; Purple: up-regulated DEGs enriched.

To further clarify the biological functions of DEGs, the “clusterprolier” in R software was performed according to the GO terms. Cellular component (CC) analysis of GO revealed that the DEGs were mainly located in “motile cilium” and 9 + 2 motile cilium (Fig. 2A). Biological process (BP) analysis showed that these DEGs were remarkably enriched in microtubule-based movement and germ cell development (Fig. 2B). Molecular function (MF) analysis showed that these DEGs were involved in the tubulin-binding pathway (Fig. 2C) (*P* < 0.05). The DEGs in three datasets (comparing NOA and OA, NOA and normal samples) showed consistent function, suggesting their potential importance for NOA.

According to the Kyoto Encyclopedia of Genes and Genomes (KEGG)^17^analysis, the up-regulated genes in datasets GSE45885 and GSE45887 were found to be enriched in pathways related to taste transduction and microRNAs in cancer. While, the up-regulated genes in GSE9210 were enriched in pathways related to complement and coagulation system, as well as steroid hormone biosynthesis. Furthermore, the down-regulated DEGs in all three datasets were significantly enriched in the pathways of glycolysis/gluconeogenesis and proximal tubule bicarbonate reclamation (Fig. 2D).

**Figure 3:**
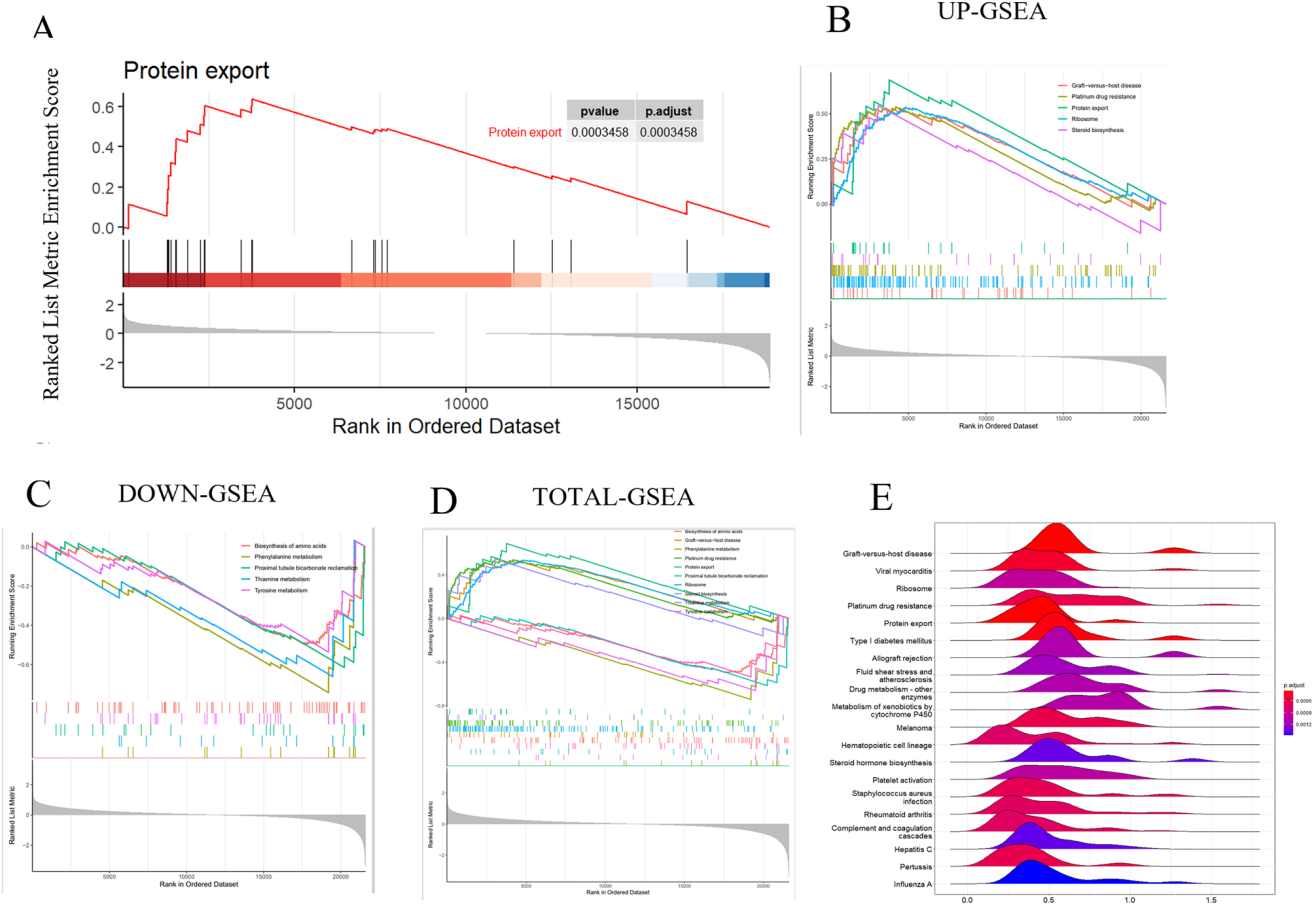
GSEA analysis of DEGs. GSEA analysis using the whole DEGs showed the protein transport pathway was significantly enriched (*P adj* < 0.001) (**A**). Enrichment plots of up-regulated DEGs (**B**, top 5), down-regulated DEGs (**C**, top 5), and total DEGs (**D**, top 10) in GSE45585, GSE45587 and GSE9210 datasets. Among them, up-regulated DEGs were enriched in graft-versus-host disease, platinum drug resistance, protein export, ribosomes and steroid synthesis (*P* < 0.05) (**B**), and those down-regulated DEGs were enriched in biosynthesis of amino acid, phenylalanine metabolism, proximal tubular bicarbonate reclamation, and thiamine and tyrosine metabolism pathways (**C**). Moreover, GSEA results indicated that these DEGs were enriched in the biosynthesis of amino acid, graft-versus-host disease pathways and metabolism pathways (**D**), and the expression distribution was demonstrated by the ridgeline plot (**E**).

To better understand the pathways in spermatogenesis arising from NOA, we combined the DEGs from the three datasets for whole-gene analysis. After removing the duplicates, 22,147 genes were applied for Gene Set Enrichment Analysis (GSEA) analysis and it was found that they were mainly involved in the protein transport pathway (*P adj* < 0.001) (Fig. 3A). Among them, up-regulated DEGs were enriched in graft-versus-host disease, platinum drug resistance, protein export, ribosomes and steroid synthesis (*P* < 0.05) (Fig. 3B), and those down-regulated DEGs were enriched in biosynthesis of amino acid, phenylalanine metabolism, proximal tubular bicarbonate reclamation, and thiamine and tyrosine metabolism pathways (Fig. 3C). Moreover, GSEA results indicated that these DEGs were enriched in biosynthesis of amino acid, graft-versus-host disease pathways and metabolism pathways (Fig. 3D), and the expression distribution was demonstrated by the ridgeline plot (Fig. 3E). In summary, these DEGs in NOA might be involved in the metabolism and the infection response pathways.

### Six hub genes were identified by protein-protein interaction (PPI) analysis

**Figure 4:**
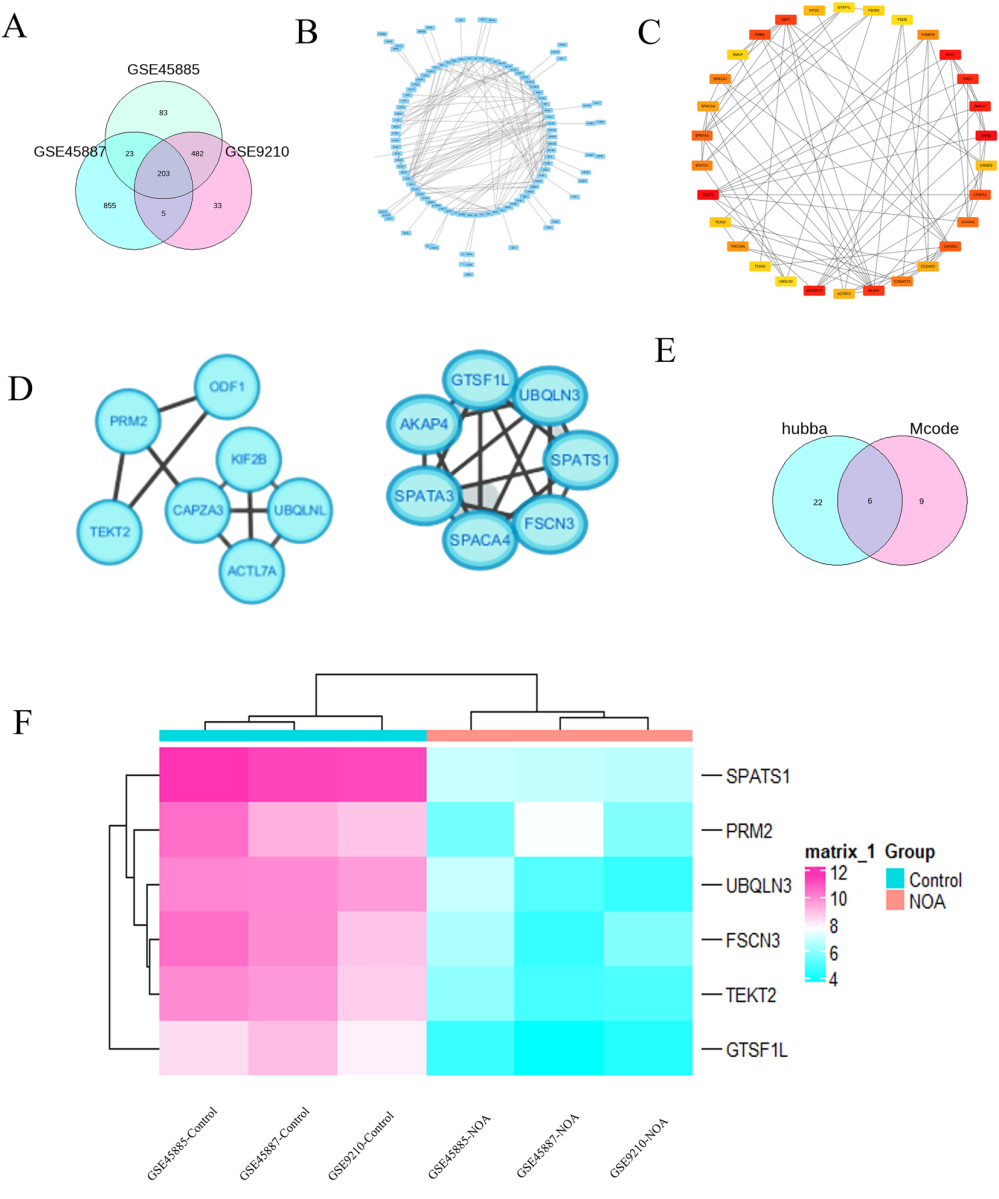
Identification of hub genes. A Venn diagram of 203 overlapping DEGs in GSE45585, GSE45587 and GSE9210 datasets (**A**). PPI network constructed by 203 DEGs in STRING database with a PPI score > 0.4 (**B**). As a result, the PPI network showed 59 interactions of the hub genes with each other, 197 nodes and 271 edges were established. Three functional modules were identified using Cytoscape software by Hubba plugin analysis (**C**) and the MCODE (**D**). Six hub genes with the highest scores in the Degree, Bottleneck, and MCC algorithms from Hubba and MCODE analysis were identified (**E**). Six identified hub genes including *PRM2, FSCN3, TEKT2, UBQLN3, SPATS1* and *GTSF1L* were all down-regulated in NOA compared to the normal samples (**F**).

A total of 203 DEGs were identified in common from the analysis using GSE45585, GSE45587 and GSE9210 datasets (Fig. 4A). In addition, to screen the critical genes in spermatogenesis, the PPI network of 203 DEGs from three datasets was constructed in the STRING database (Fig. 4B). As a result, the PPI network showed 59 interactions of the hub genes with each other (the average local clustering coefficient was 0.327 and the PPI enrichment *P* value was 1.0e-16), 197 nodes and 271 edges were established (Fig. 4B) and three functional modules were identified using Cytoscape software by Hubba plugin analysis (Fig. 4C) and the MCODE (Fig. 4D). In total, six spermatogenesis-related genes including protamine 2(*PRM2*)*,* Fascin Actin-Bundling Protein 3 (*FSCN3*)*,* tektin 2 (*TEKT2*), ubiquilin 3 (*UBQLN3*), spermatogenesis associated, serine-rich 1 (*SPATS1*) and gametocyte specific factor 1-like (*GTSF1L*) with the highest scores in the Degree, Bottleneck, and MCC algorithms were identified as the hub genes (Fig. 4E). Interestingly, six identified hub genes were all down-regulated in NOA compare to the normal and OA samples (Fig. 4F).

### Three hub gene expressions were validated in the dataset GSE145467 by in-silico analysis

**Figure 5:**
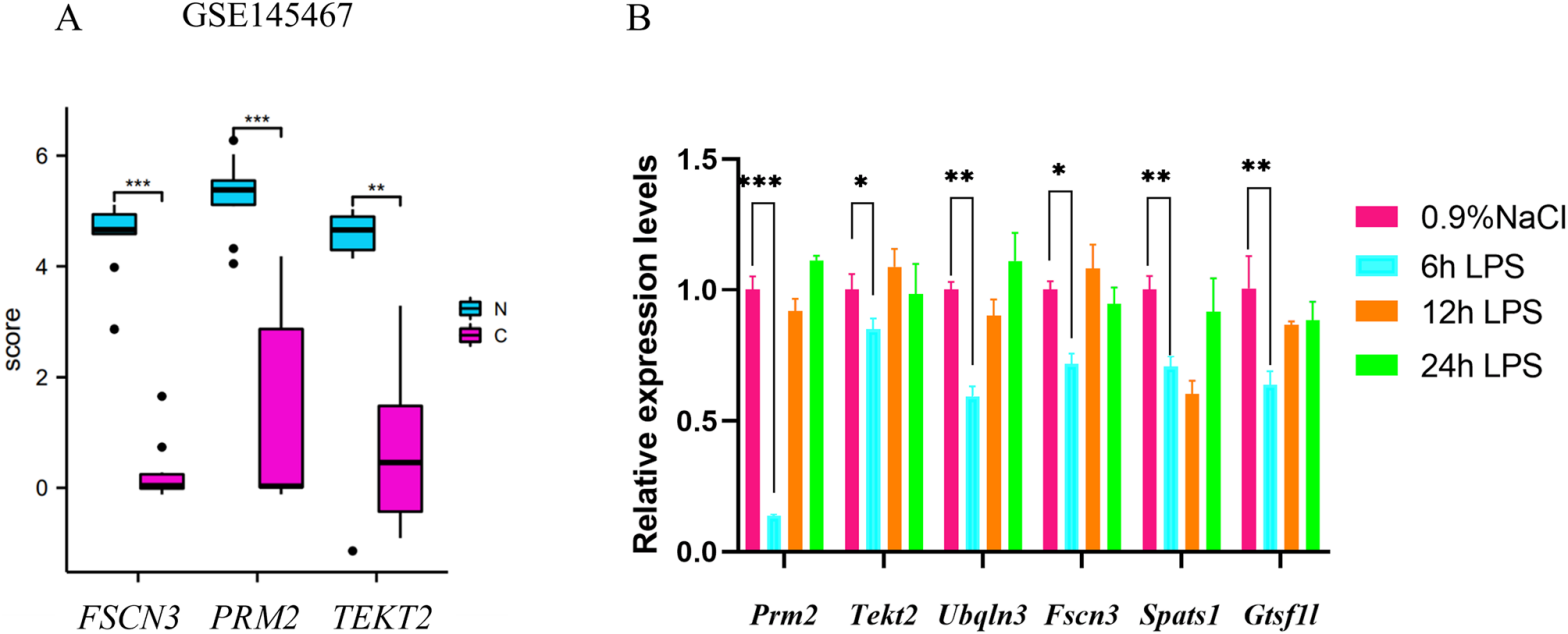
Independent validation of the expression of hub genes in the GSE145467 dataset (**A**) and testes of lipopolysaccharide-(LPS-) induced acute orchitis mice (n = 4 animals/group) by qRT-PCR (**B**). *β-actin* was used as an internal control. *P < 0.05; **P < 0.01; ***P < 0.001.

To further confirm the analysis result of hub gene identification from three gene expression omnibus (GEO) datasets, we examined the expression of six potential hub genes in the GSE145467 dataset. In the NOA group, *PRM2, FSCN3,* and *TEKT2* were found to be down-regulated (*P* < 0.05) (Fig. 5A). This result was consistent with the findings from three GEO datasets analysis, indicating that these genes may play a crucial role in spermatogenesis. However, the GSE145467 dataset did not show any expression of *UBQLN3, SPATS1*, and *GTSF1L*.

### Six hub genes were down-regulated in the testis of LPS-induced acute orchitis mouse

The NOA phenotype is complex and caused by disturbed/disrupted spermatogenesis due to testicular trauma, neoplasia, and/or orchitis/epididymitis^18^. Chronic immune-mediated orchitis can cause acquired NOA and lead to spermatogenetic failure in dogs^19^. Thus, to better understand the differential expression and function of the hub genes in spermatogenesis, we induced an acute orchitis mouse model by intraperitoneal (i.p.) LPS injection, which showed impaired spermatogenesis according to the protocol previously reported^20^. After 6 h induction by i.p. LPS injection, the mouse testicular morphology was examined. Fewer germ cells were observed and the spermatogenic cells were disorganized and disrupted compared to the control group (injected with 0.9% NaCl) (Supplementary Fig. 3). The results of the analysis based on the sperm parameters indicated a significant decrease in sperm viability (*P* < 0.01), density (*P* < 0.01), amplitude of lateral head displacement (*P* < 0.01), percentage of motile sperm (*P* < 0.05) and curve-line velocity (*P* < 0.05) (Supplementary Fig. 4). However, the testicular morphology showed progressive recovery after 12 h, with no apparent morphological changes observed at 24 h following LPS injection (Supplementary Fig. 3). There were no significant differences in sperm viability and motility compared to the control group (Supplementary Fig. 4). We also examined the expression of protamine 1 (*Prm1*), which is a molecular marker of spermatids, and discovered that it was down-regulated in the testes of mice induced with LPS for 6 h compared to the control group (Supplementary Fig. 5). These findings suggest that abnormal spermatogenesis occurs in mice with acute orchitis 6 h after LPS injection. Consequently, we utilized the acute orchitis mouse model to verify hub genes in cases of impaired spermatogenesis after 6 h of LPS injection. Subsequently, we examined the expression of identified potential hub genes including *Prm2, Fscn3, Tekt2, Ubqln3, Spats1* and *Gtsf1l* in the testes of acute orchitis mice (4 animals/group) by qRT-PCR. Compared to the control group, the expression of six hub genes was all down-regulated in the testes of acute orchitis mice at 6 h after LPS injection (Fig. 5B). This finding provided strong support for our bioinformatics analysis based on three expression datasets.

### The long noncoding RNA (lncRNA)-microRNA (miRNA)-messager RNA (mRNA) and transcription factor (TF)-miRNA-mRNA regulatory networks of *PRM2, FSCN3* and *TEKT2* genes

**Figure 6:**
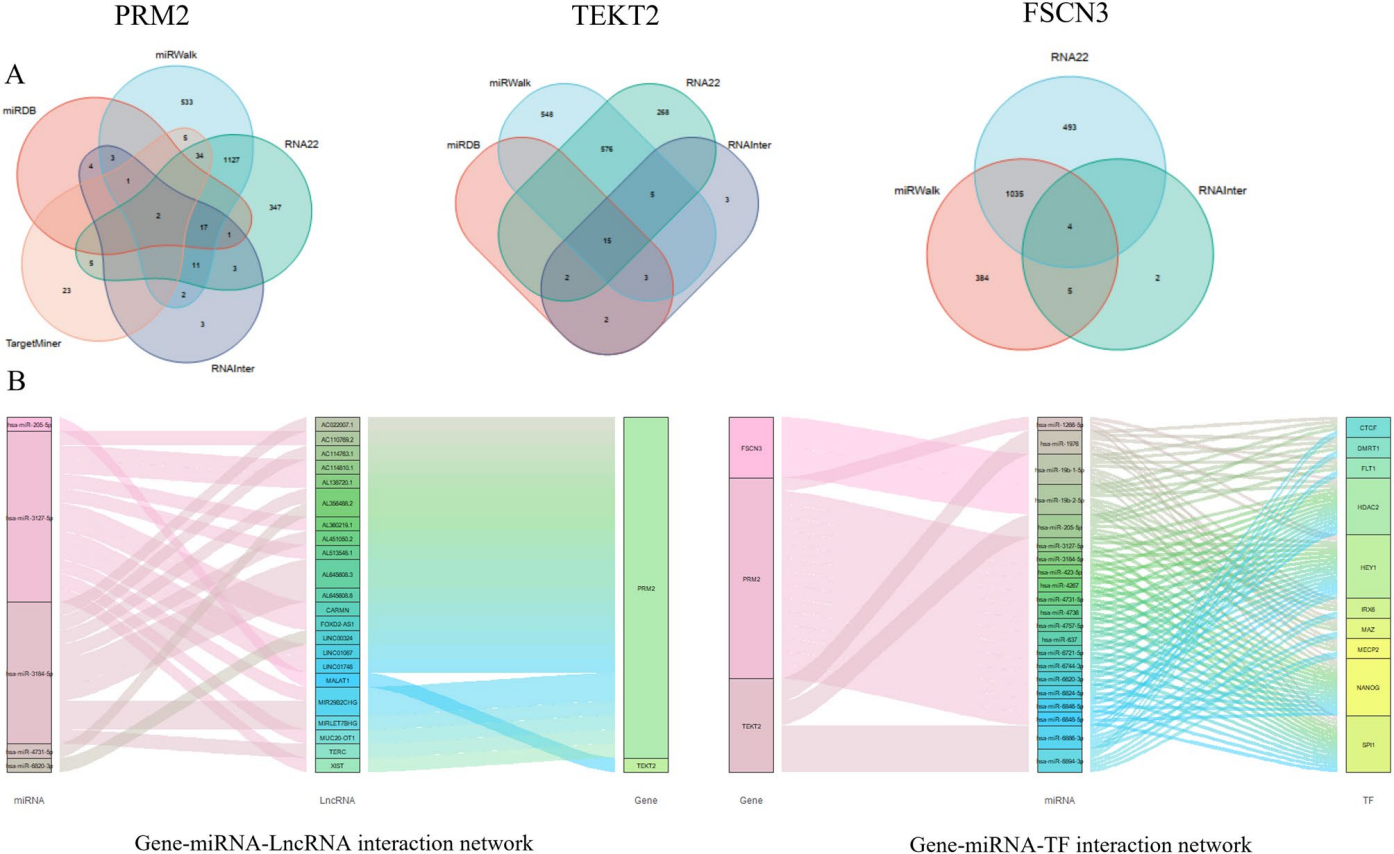
Prediction of the regulatory networks of three hub genes. Venn maps showed the predicted miRNAs of *FSCN, PRM2,* and *TEKT2* using ENCORI, miRDB, miRWalk, RNA22, RNAInter, TargetMiner, TargetScan, NPInter, miRTarBase databases (**A**). Sankey diagram represents the networks of mRNA-miRNA-lncRNA (**B**, left). LncRNA was predicted using the ENCORI database. Sankey diagram represents the networks of mRNA-miRNA-TF (**B**, right). TF was predicted by the iRegulon plug-in in Cytoscape.

To reveal the functions of three hub genes in spermatogenesis, we constructed the lncRNA-miRNA-mRNA and TF-miRNA-mRNA regulatory networks of *PRM2, FSCN3* and *TEKT2* genes. Firstly, nine lncRNA-miRNA-mRNA databases including ENCORI, miRDB, miRWalk, RNA22, RNAInter, TargetMiner, TargetScan, NPInter and miRTarBase databases were screened by R software. Then, the predicted regulatory miRNAs were identified from the intersection of screening results of 9 databases (Fig. 6A). In addition, we predicted the TFs of the hub gene through the iRegulon plugin of Cytoscape. Finally, lncRNA-miRNA-mRNA and TF-miRNA-mRNA networks were plotted as a Sankey diagram and visualized by Cytoscape (Fig. 6B). A variety of TFs and noncoding RNAs were identified to regulate the *PRM2, FSCN3* and *TEKT2* expression and might be associated with spermatogenesis. Among them, miRNAs (has-miR-3127-5p and has-miR-3184-5p), lncRNAs (AL356488.2 and AL645608.3) and TFs (NANOG and HEY1) as the key regulatory factors of *PRM2, FSCN3* and *TEKT2*, were predicted significantly associated with male infertility.

### Expression and methylation of six hub genes in the testicular germ cell tumors (TGCTs) from the cancer genome atlas (TCGA) database

**Figure 7:**
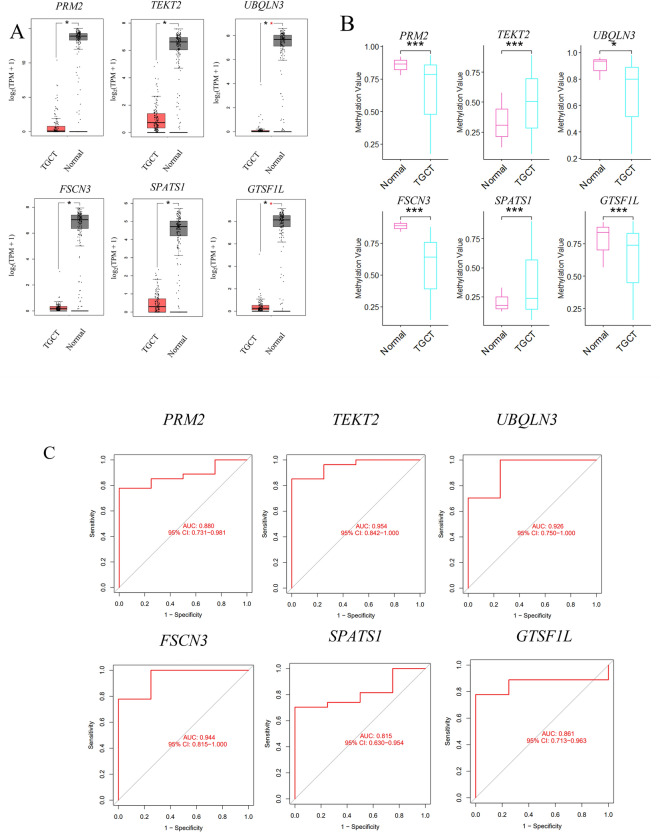
Analysis of six hub genes in TGCTs from the TGCA database. Boxplots of six hub genes expression in TGCTs from TGCA database (**A**). *PRM2, TEKT2, FSCN3, UBQLN3, SPATS1* and *GTSF1L* were significantly down-regulated in TGCT samples compared to normal controls. *P < 0.05. Methylation levels of 6 hub genes in TGCT and control groups (**B**). The methylation levels of *PRM2, TEKT2, FSCN3, UBQLN3, SPATS1* and *GTSF1L* were significantly changed in TGCT samples compared to normal controls (*P* < 0.05). Among them, the methylation levels of *PRM2, FSCN3, UBQLN3* and *GTSF1L* were significantly increased in TGCT compared to normal samples, while the mean methylation levels of *TEKT2* and *SPATS1* were decreased (*P* < 0.05). ROC analysis showed six hub genes showed good prognostic value with an area under the curve (AUC) ≥ 0.7 (**C**). *TEKT2* (AUC = 0.954, 95% CI: 0.842–1.000; *PRM2* (AUC = 0.880, 95%CI: 0.731–0.981); *FSCN3* (AUC = 0.944, 95% CI: 0.815–1.000); *UBQLN3*(AUC = 0.926, 95% CI: 0.750–1.000); *SPATS1*(AUC = 0.815, 95% CI: 0.630–0.954); *GTSF1L* (AUC = 0.861, 95% CI: 0.713–0.963).

TGCTs are the most common solid malignancy of adolescents and young men^21^. However, the pathogenesis and underlying mechanisms of TGCT remain unclear. The clinical factors that are predisposed to the development of TGCT include cryptorchidism and testicular microlithiasis, as well as infertility^22^. The impaired expression of antiapoptotic genes is thought to be involved in idiopathic fertility defects and the onset and progression of testicular germ cells^23^. The genetic factors, as important contributors to TGCT, will be helpful for early diagnosis. Targeting these genes will improve the efficiency of the treatment of this cancer. Since the hub genes identified from our analysis were associated with spermatogenesis, thus, we would like to explore their expressions in the TGCT samples from the TCGA database using the Gene Expression Profiling Interactive Analysis (GEPIA) analysis. It was found that these six hub genes were significantly down-regulated in TGCTs compared to normal samples (*P* < 0.05) (Fig. 7A), indicating that the dysregulated expression of these hub genes in TGCT might play an essential role in tumor occurrence, progression, and metastasis and be the potential biomarkers for diagnosis.

In spermatogenesis, the dysregulation of epigenetic modifications, in particular the methylation of sperm genomic DNA, may function in the development of many diseases^24^. We analyzed the methylation levels of six hub genes in DiseaseMeth. Our findings revealed that in TGCT patients, the methylation levels of *PRM2, FSCN3, UBQLN3* and *GTSF1L* were significantly higher than those in the normal group (*P* < 0.05, Fig. 7B). Conversely, the average methylation levels of *TEKT2* and *SPATS1* were lower (Fig. 7B). This led us to speculate that the highly methylated genes *PRM2, FSCN3, UBQLN3* and *GTSF1L* in testicular cells may regulate gene expression and have an impact on testicular fertility.

### Hub genes were predicted as the potential TGCT biomarkers by ROC analysis

To assess the diagnostic effectiveness of these six hub genes, the receiver operating characteristic (ROC) analysis was performed by the pROC R package (version 1.16.2). All hub genes displayed good prognostic value with an area under the curve (AUC) ≥ 0.7 (Fig. 7C). Among them, the *TEKT2* gene showed the highest AUC (AUC = 0.954, 95% CI:0.842–1.000) (Fig. 7C). In conclusion, the ROC curves and the AUC values indicated that the gene expression levels of six hub genes (*PRM2, TEKT2, FSCN3, UBQLN3, SPATS1* and *GTSF1L*) could discriminate TGCT and normal samples (Fig. 7C). This result suggested that these genes have a high potential to serve as TGCT biomarkers.

## Discussion

In the present study, we used a bioinformatics strategy and publicly available databases to identify hub genes in NOA which result in abnormal spermatogenesis. GSE45887, GSE45885 and GSE9210 datasets were used to identify the hub genes and evaluate the expression level. As a result, we identified 203 DEGs including 34 up-regulated and 169 down-regulated between NOA and normal spermatogenesis samples including OA and normal control. The majority of them are associated with spermatogenesis and male infertility. DEGs were mainly involved in microtubule motility and the process of cell growth, especially associated with cilia motility and sperm flagella. These pathways have been related to sperm motility, testis structure, and sperm development^25–27^. The bioinformatics analysis result was independently verified in a validation dataset of NOA *in-silico* and the acute orchitis mouse testis with impaired spermatogenesis experimentally.

Our study has identified 6 hub genes from the gene expression profiles of three datasets between NOA and normal samples. Further analyses validated the down expression of 6 hub genes in silico and their dysregulation was also confirmed by qRT-PCR experiment in the testis of LPS-induced acute orchitis mice which showed abnormal spermatogenesis. Moreover, we found that *PRM2, TEKT2,* and *FSCN3* were significantly down-regulated not only in NOA samples but also in TGCT compared to their expression in normal samples. The result indicated that these three genes could play an important role in spermatogenesis and also be potential biomarkers for the diagnosis of TGCT. In addition, we found these three genes were enriched in several biological pathways including chromosome condensation, actin filament bundle assembly, and axonemal dynein complex assembly by GO and KEGG analyses. The regulatory network of TF-miRNA-mRNA and lncRNA-miRNA-mRNA was predicted and revealed that has-miR-3127-5p and has-miR-3184-5p might be the regulator of *PRM2、FSCN3* and *TEKT2* and play a critical role in azoospermia and TGCT. The miRNA of has-miR-3127-5p regulating the target key genes of tumor mutation burden (TMB), has a potential effect on the therapeutic responses in cancers^28^. In addition, serum has-miR-3184-5p was predicted as a diagnosis biomarker for multiple cancer types^29^. Both miRNAs have not been identified with spermatogenesis or testis development yet.

One of the hub genes was identified as protamine 2 (*PRM2*). Protamine is an arginine-rich nuclear protein that is specifically localized in the nucleus of sperm to prevent mutations in the genome of sperm from internal and external environments ^30^. In the process of sperm formation, approximately 85% of sperm histones are replaced by *PRM1* and *PRM2* for chromatin remodeling^31^, which is necessary for spermatozoa to perform normal functions. The protamine replacement leads to the termination of transcription in spermiogenesis and causes changes in the shape of the sperm nucleus and limits the sperm swimming speed by affecting sperm flow efficiency^32^. The changes in one or both of these two protamine proteins can lead to abnormal sperm chromatin, increase the chance of double-strand break of sperm DNA, induce oxidative stress, lead to decreased motility, and ultimately lead to male infertility^33,34^. Loss of PRM2 initiates an oxidative stress-mediated destruction cascade during epididymal sperm maturation^35^. Low levels of PRM2 may be associated with morphological abnormalities, initiation of the apoptotic pathway, and decreasing sperm motility in the study using the semen of infertile men^36^. Here, in our current study, the down-regulation of *PRM2* was identified as closely related to NOA and TGCT.

Fascins are well-conserved actin-bundling proteins ^37^. In vertebrates, there are three members of FSCN, namely FSCN1, FSCN2, and FSCN3. Among them, FSCN1 is widely expressed in the nervous system and mesenchymal tissues^37–40^ and crosslinking actin microfilaments into tight, rigid, and parallel bundles^41^. Knockout of *Fscn1* in mice resulted in half neonatal lethality and reduced body weight of the surviving mice^42^. The increased FSCN1 levels can enhance the migration and invasion of multiple cancers and be associated with poor patient prognosis^43^. The expression of miR-145 in breast and ovarian cancer cells affects cell migration and inhibits EMT by targeting FSCN1^44^. FSCN2 has been found in retinal photoreceptor cells and its splicing variants might be related to retinitis pigmentosa and hearing loss^45,46^. While *FSCN3* is a testis-specific expressed gene, mainly expressed in elongated spermatids during late spermiogenesis^47^. During spermiogenesis, round spermatids are remodeled into the fusiform shape of mature spermatozoa, and these morphological changes are closely correlated with a reorganization of microfilaments and microtubules in the head and tail regions of elongating spermatids^48^, accompanied with altered expression of specialized actin- and tubulin-associated proteins. The actin-bundling proteins, namely, alpha-actinin, fimbrin/plastin, fascin, and espinis, are thought to play an essential role in the formation or support of dynamic cell structure. Qu et al. Found that dietary vitamin E supplements in sheep could cause the differential expression of Fscn3 and be beneficial to spermatogenesis^49^. However, the *Fscn3*^*-/-*^ knockout mice through CRISPR/Cas9 gene-editing are fertile with normal testis development, and it indicated that *Fscn3* is not required for mouse spermatogenesis^50^.

In our study, we also found *TEKT2* as one of the hub genes for spermatogenesis. Tektins are skeleton proteins for microtubules in cilia, flagella, basal bodies, and centrioles^51^. Five members of Tektins have been identified in humans including TEKT1, TEKT2, TEKT3, TEKT4, and TEKT5^52–55^. Human TEKT2 (also named Tektin-t & h-tekB1) is present in the principal piece of spermatozoa and plays a critical role in the formation and development of the cilia or flagella of spermatozoa^56^. A previous study reported that the mutation in the *TEKT2* gene can cause defects in flagella activity, which could have a hazardous effect on spermatozoa motility, leading to male infertility^57^. *TEKT2* was also found down-regulated in dysfunctional spermatozoon after cryopreservation^58^.

Moreover, we investigated the hub gene expression in testicular tumors TGCT which showed impaired spermatogenesis. Interestingly, three hub genes including *PRM2, FSCN3* and *TEKT2* were also down-regulated in TGCT, consistent with the results from the NOA study. These three genes could become the potential diagnostic biomarkers for TGCT. However, the specific molecular mechanism needs to be investigated further.

Although we identified 6 hub genes for spermatogenesis, our results still have some limitations. Firstly, this *in-silico* analysis only included samples from publicly available databases. Secondly, the results lack verification in human TGCT samples. Taken together, our analysis results will provide new insight into the mechanism of spermatogenesis and TGCT.

In summary, we discovered DEGs and screened the hub genes involved in spermatogenesis through a relatively systematic bioinformatics strategy. *PRM2, FSCN3* and *TEKT2* were identified as hub genes for spermatogenesis by bioinformatic and experimental studies. In addition, we found that a variety of noncoding RNAs and TFs might regulate the hub gene expression and they play an essential role in spermatogenesis. Together, our *in-silico* method identified several hub genes and their regulatory factors in impaired spermatogenesis arising from NOA, TGCT, and orchitis, and these factors can act as potential therapeutic targets for male infertility.

## Materials and methods

### Data collection and preprocessing

Three gene expression datasets (GSE45887, GSE45885 and GSE9210) based on the platform GPL6244 and GPL887 (Affymetrix Human Gene Expression Array) were downloaded from the publicly available GEO database (https://www.ncbi.nlm.nih.gov/geo/). The dataset GSE145467 from platform GPL4133 was obtained as a verification set. Detailed information on the data types is listed in Table 1. The inclusion criteria were set as follows: (1) samples obtained from human gene expression arrays; (2) samples from the patients with NOA and normal spermatogenesis controls; (3) there are at least 20 samples in each dataset. The gene expression profile (FPKM, fragments per kilobase of transcript per million fragments mapped) and clinical information of patients with TGCT were obtained from the TCGA database (https://portal.gdc.cancer.gov/) and analyzed by GEPIA.

R packages (http://www.bioconductor.org, http://www.github.com) were used for data processing. The GEOquery package (version 2.64.2) and stringr package (version 1.4.1) in R software were used to download and clean the GEO datasets, respectively. After removing the missing values, all the data were grouped. The expression profile FPKM was converted to TPMs (transcripts per kilobase million) with log2 (FPKM + 1) for further analysis.

### Identification of DEGs

The principal component analysis (PCA) was carried out using the FactoMineR package (version 2.6), and the data transformation was performed by the factoextra package (version 1.0.7) for DEG analysis. The pheatmap package (version 1.0.12) was used to visualize the data. The limma package (version 3.52.1) was subsequently used for DEG identification. DEGs were screened based on the criteria of *P* *adj* < 0.05 and |log_2_(fold change, FC)|> 1.0. Finally, the identified DEGs were visualized using the ggplot2 package (version 3.3.6) and the intersection of DEGs was visualized by constructing the Venn diagram.

### GO and KEGG functional enrichment analysis of DEGs

The biological function of the DEGs was enriched using GO function enrichment and KEGG pathway analysis with the clusterprofiler package in R (version 4.4.1) and the enrichGO function in the enrichplot package (version 1.16.1). The criteria for enrichment were set as *Q-*value = 0.01. *Q-value* < 0.05 was considered statistically significant. The GO annotation of DEGs was subjected to three categories including biological process (BP), cellular component (CC), and molecular function (MF).

### GSEA analysis of expressed genes in GSE45887, GSE45885 and GSE9210 datasets

GSEA R Package was applied to determine the difference in the biological pathways of the DEGs according to the enrichment score. The KEGG gene profile “c2.cp.kegg.v7.4.symbols.gmt” from the Molecular Signatures Database (MSigDB) database was downloaded to carry out the functional enrichment analysis. *Q-*value < 0.05 was considered statistical differences between distinct groups.

### PPI construction and hub gene identification

PPI networks were constructed with DEGs by the Search Tool for the Retrieval of Interacting Genes (STRING, https://cn.string-db.org/) with a PPI score > 0.4. Subsequently, the functional modules of the PPI networks were visualized by Cytoscape software (version 3.7.1, https://www.cytoscape.org/). The Bron–Kerbosch algorithm (MCC) in the Hubba plug-in (node score cut-off = 0.2) and Molecular Complex Detection (MCODE) plugin in Cytoscape (node degree = 2, score cluster = 0.2, K-core = 2, Max. Depth = 100) were used to analyze the subnetworks within the PPI network. The intersection of the two outcomes was used to detect hub genes for NOA.

### LPS-induced acute orchitis mouse

Sixteen *Balb/c* male mice (5 weeks old, weighing 26 ± 2 g) were purchased from the Vital River Laboratory Animal Technology Ltd., Beijing, China [Animal Certificate Number: SCXK(jing)2021–0006]. All animal studies were approved by the Animal Ethics Committee of Inner Mongolia Medical University (Ethics Approval Number: YKD2019143) and carried out in accordance with the relevant guidelines and regulations for the use and care of animals. This study was performed in compliance with the ARRIVE guidelines. All mice were housed in a room under standardized conditions at 21–25 °C, 45–65% humidity, and a 12-h light–dark cycle. The mice were randomly divided into four groups of 4 animals each: (I) intraperitoneal (i.p.) saline group (0.9% NaCl), (II) i.p. 6 h after LPS (3.3 mg/kg) injection group (6 h LPS), (III) i.p. 12 h after LPS (3.3 mg/kg) injection group (12 h LPS) and (IV) i.p. 24 h after LPS (3.3 mg/kg) injection group (24 h LPS). LPS-induced acute orchitis protocol was performed according to the procedure originally described by Haley and McCormick^20^. All mice were anesthetized with sodium pentobarbitol (60 mg/kg) and sacrificed. Then the testes were removed for further study. One of a pair of testes from each group was fixed in 10% formalin for histological analysis, the other one was stored at -80 °C for RNA isolation. The epididymis was collected for sperm analysis.

### Hematoxylin and eosin (HE)staining

The formalin-fixed testes were washed in a progressively decreasing concentration of ethanol, embedded in paraffin, and sectioned by a rotary microtomy (Minux® S700, RWD, China). HE staining was performed subsequently. The pictures were taken using an Olympus microscope IX73 with CellSens Standard Software (Olympus, Japan).

### Sperm morphology analysis

The sperm was released from the epididymis in 0.9% NaCl and kept at 37 °C for 5 min. Then 10 μL solution was placed onto a pre-warmed slide (37 °C) for morphological examination. The sperm motility, the number of motile sperm, sperm density and VSL were analyzed by the computer-assisted sperm analysis system (ML-608JZ, Nanning Songjing Tianlun Biotechnology Co., Ltd). At least 100 sperm were then video-captured in different fields.

### RNA isolation and quantitative reverse-transcription polymerase chain reaction (qRT-PCR)

The total RNA from the testes of LPS-induced acute orchitis mice was extracted by RNA-easy Isolation Reagent (VazymeBiotech, Nanjing, China). RNA concentration and purity were measured using a NanoDrop™ 2000C spectrophotometer (Thermo Fisher Scientific, USA). Subsequently, 1 μg RNA was reversely transcripted into cDNA with HiScript® II Q RT SuperMix for qPCR (Vazyme, Nanjing, China). qRT-PCR with TB Green® Premix Ex Taq (Tli RNase H Plus) (Takara, Japan) and transcript cDNA was performed to detect mRNA expression using the ABI 7500fast instrument (Applied Biosystems, USA). All the primers were synthesized by Sangon Biotech (Sangon, Shanghai, China), and the house-keeping genes beta-actin (*β-actin*) and glyceraldehyde-3-phosphate dehydrogenase (*Gapdh*) were used as internal controls (Table 2). The relative expression level of mRNA was calculated by the 2^−∆∆Ct^ method and three replicate experiments were performed.

**Table 2:** Primers used for PCR amplification.

Gene symbol	Forward primers (5′-3′)	Forward primers (5′-3′)	PCR product size (bps)	Annealing temperature (°C)
*Prm2*	GTCCCTCCTCCTCCAATCCA	CCTCGCGTTCATGGTCTTGT	238	60
*Tekt2*	AGGAGTTCTCAGACCTGGGC	GAACCCCTTAGGCCTGTTCC	432	59
*Fscn3*	AGCCCCATCTACCACAGCTA	CCAGAAGGATCCACCCCTGA	79	60
*Ubqln3*	TGCTCAATGGTGTGCCTGAT	ACAGCGCAGACATTGCTTTG	198	57
*Spats1*	ACAACAGCCTCGGGAAGAAG	GAGAAGTTCACCGGAGGCAA	267	60
*Gtsf1l*	GCTCTTGTACCTGCCTGAGT	CCCCGTGACTCTTGGATGTC	192	58
*Prm1*	ATCCACCAAACTCCTGCCTG	ACAGGCGGCATTGTTCCTTA	115	59
*Gapdh*	AGGTCGGTGTGAACGGATTTG	TGTAGACCATGTAGTTGAGGTCA	621	59
*β-actin*	AGTGTAACGTTGACATCCGT	AGCTCAGTAACAGTCCGCCTA	296	59

### Construction of TF-miRNA-mRNA and lncRNA-miRNA-mRNA regulatory network

To identify the regulatory miRNAs and lncRNAs of hub genes with abnormal spermatogenesis, nine gene prediction networks (ENCORI, miRDB, miRWalk, RNA22, RNAInter, TargetMiner, TargetScan, NPInter, miRTarBase) were screened using the ggsci (version 1.5.6) and venn (version 1.16.5) packages in R software. The transcription factors (TFs) of hub genes were predicted by the iRegulon plugin in Cytoscape. Finally, the interactive Sankey diagram was created using the ggraph package (version 2.0.6) and the igraph package (version 1.3.4).

### Hub genes expression and methylation analysis in the TGCT from the TCGA database

To evaluate gene expression in testicular germ cell tumors and comprehensively discover the importance of genes in male infertility, we used the GEPIA database (http://gepia.cancer-pku.cn/) which contains RNA-seq data from 156 tumors and 100 normal samples in the TGCT-TCGA database and GTEx projects. The expression of key genes was searched with the parameter to |Log_2_FC| Cutoff = 1, *p*-value Cutoff = 0.01, Jitter Size = 0.4 in GEPIA.

MEXPRESS (https://mexpress.be/) is a database that visualizes the relationship between clinical information of patients—methylation—gene expression in the TCGA database, which we chose for analysis to understand the regulatory relationship of key genes. The filter criteria were set as follows: (I) Platform selection: 450 k (Illumina Infinium HumanMethylation450 BeadChip); (II.) Statistical analysis methods: T-test; (III.) Screening criteria: *P* < 0.05; (IV.) Absolute methylation difference > 0.2.

### ROC curve analysis

To predict the probability of hub genes as diagnostic biomarkers of TGCT, ROC curve analysis which is powerful in the diagnostic evaluation was carried out via the pROC R package (version 1.17.6). The area under the ROC curve (AUC) > 0.60 was considered acceptable for the prediction ability of the model, and an AUC > 0.75 was considered to indicate a good predictive value.

### Statistical analysis

All statistical analyses were performed using the R software and GraphPad Prism (8.0.2). The Wilcoxon test was utilized to compare the two groups, while the Student's t-test was used for methylation level analysis. The one-way ANOVA method was used and followed by a post-hoc test among three groups. A *P*-value of < 0.05 was considered statistical significance.

### Supplementary Information

Supplementary Figure 1.

Supplementary Figure 2.

Supplementary Figure 3.

Supplementary Figure 4.

Supplementary Figure 5.

Supplementary Legends.

## Data Availability

The datasets generated and/or analyzed for this study can be found in the GEO repository. GSE45885 is available at: https://www.ncbi.nlm.nih.gov/geo/query/acc.cgi?acc=GSE45885, GSE45887 is available at: https://www.ncbi.nlm.nih.gov/geo/query/acc.cgi?acc=GSE45887, GSE9210 is available at: https://www.ncbi.nlm.nih.gov/geo/query/acc.cgi?acc=GSE9210, GSE145467 is available at: https://www.ncbi.nlm.nih.gov/geo/query/acc.cgi?acc=GSE145467.
