# Supplementary figures and images for: Identification of hub genes associated with spermatogenesis by bioinformatics analysis
Source: Sci Rep. 2023 Oct 27;13:18435. doi: 10.1038/s41598-023-45620-3 (PMC10611713; doi:10.1038/s41598-023-45620-3)

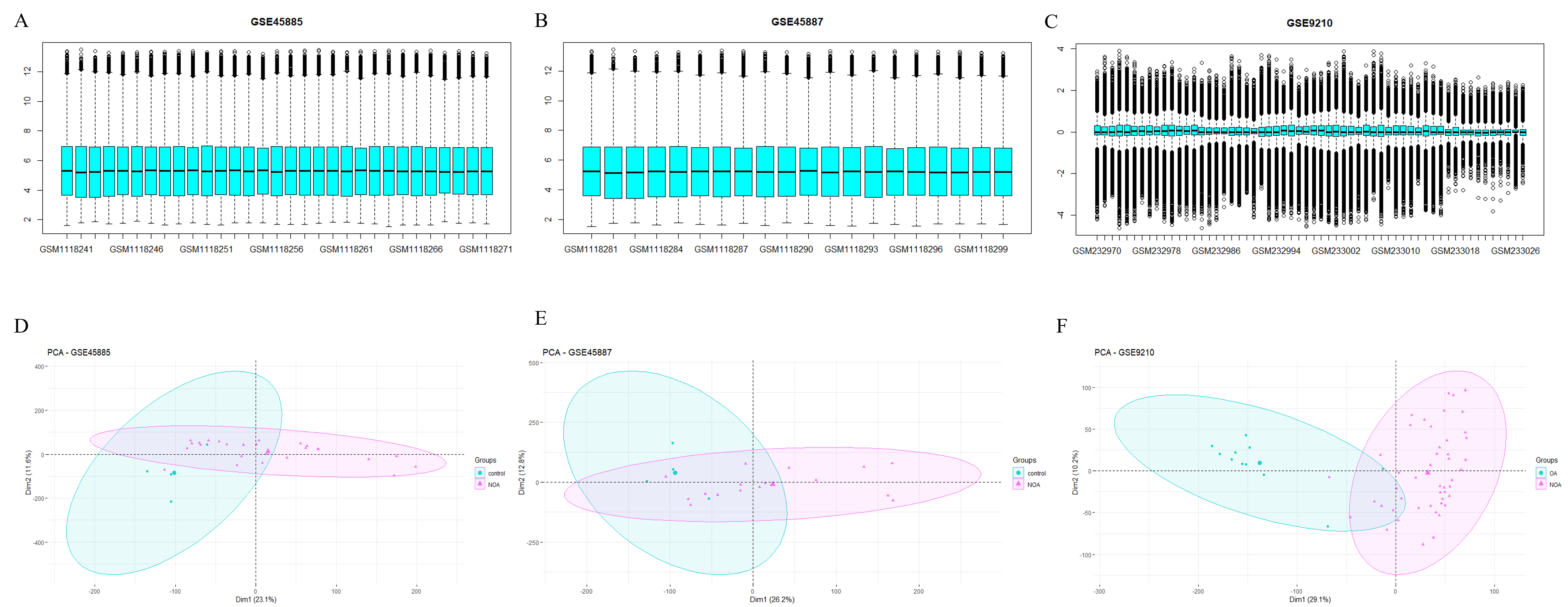

Supplement: Supplementary file 1 — Supplementary Figure 1. [file 41598_2023_45620_MOESM1_ESM.tif]

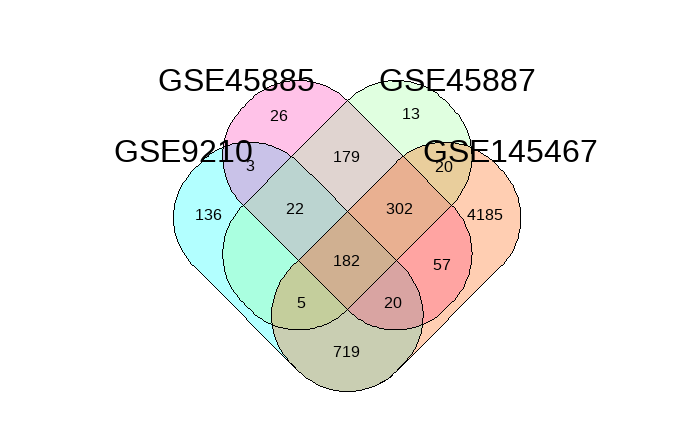

Supplement: Supplementary file 2 — Supplementary Figure 2. [file 41598_2023_45620_MOESM2_ESM.tiff]

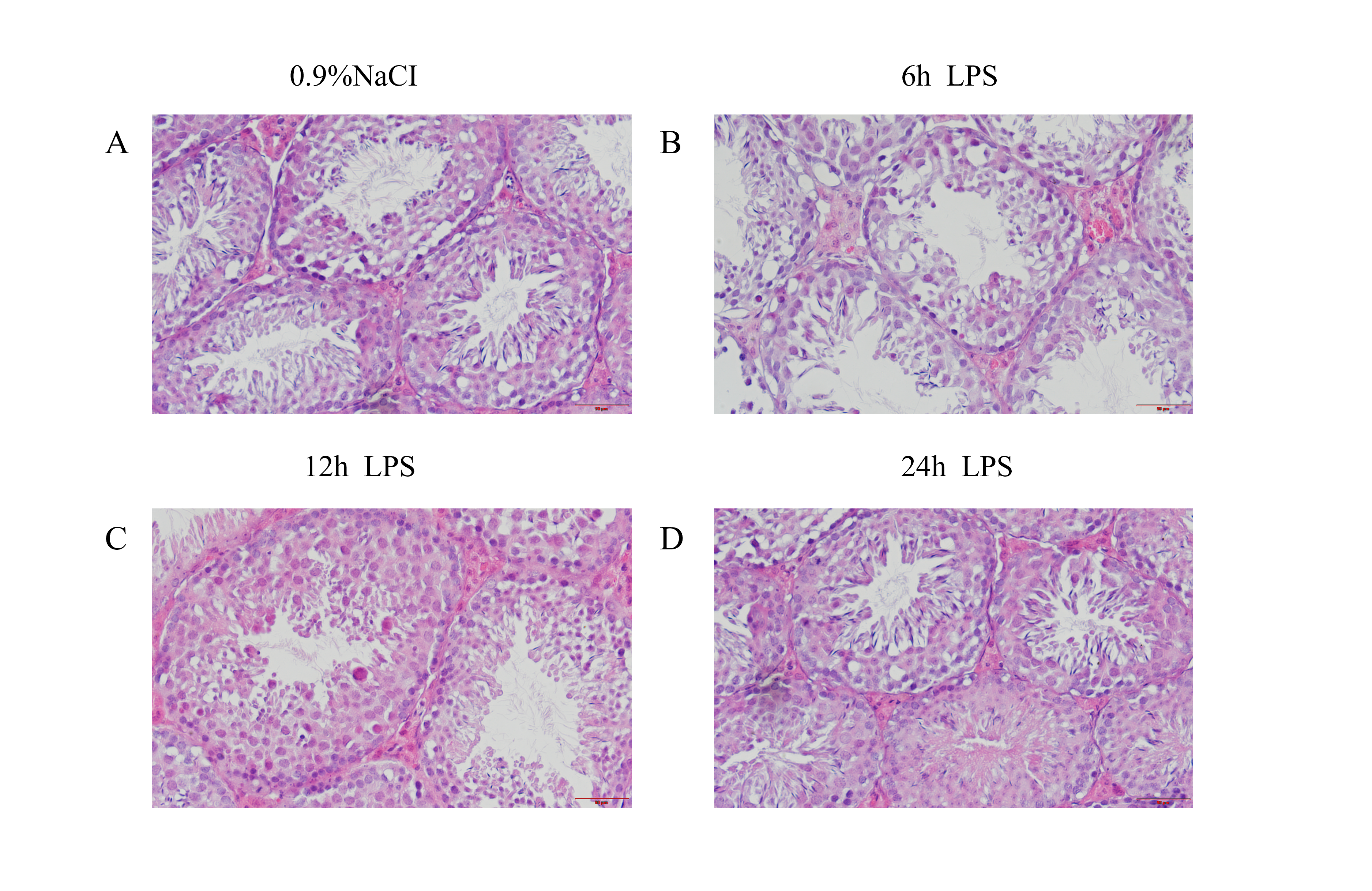

Supplement: Supplementary file 3 — Supplementary Figure 3. [file 41598_2023_45620_MOESM3_ESM.tif]

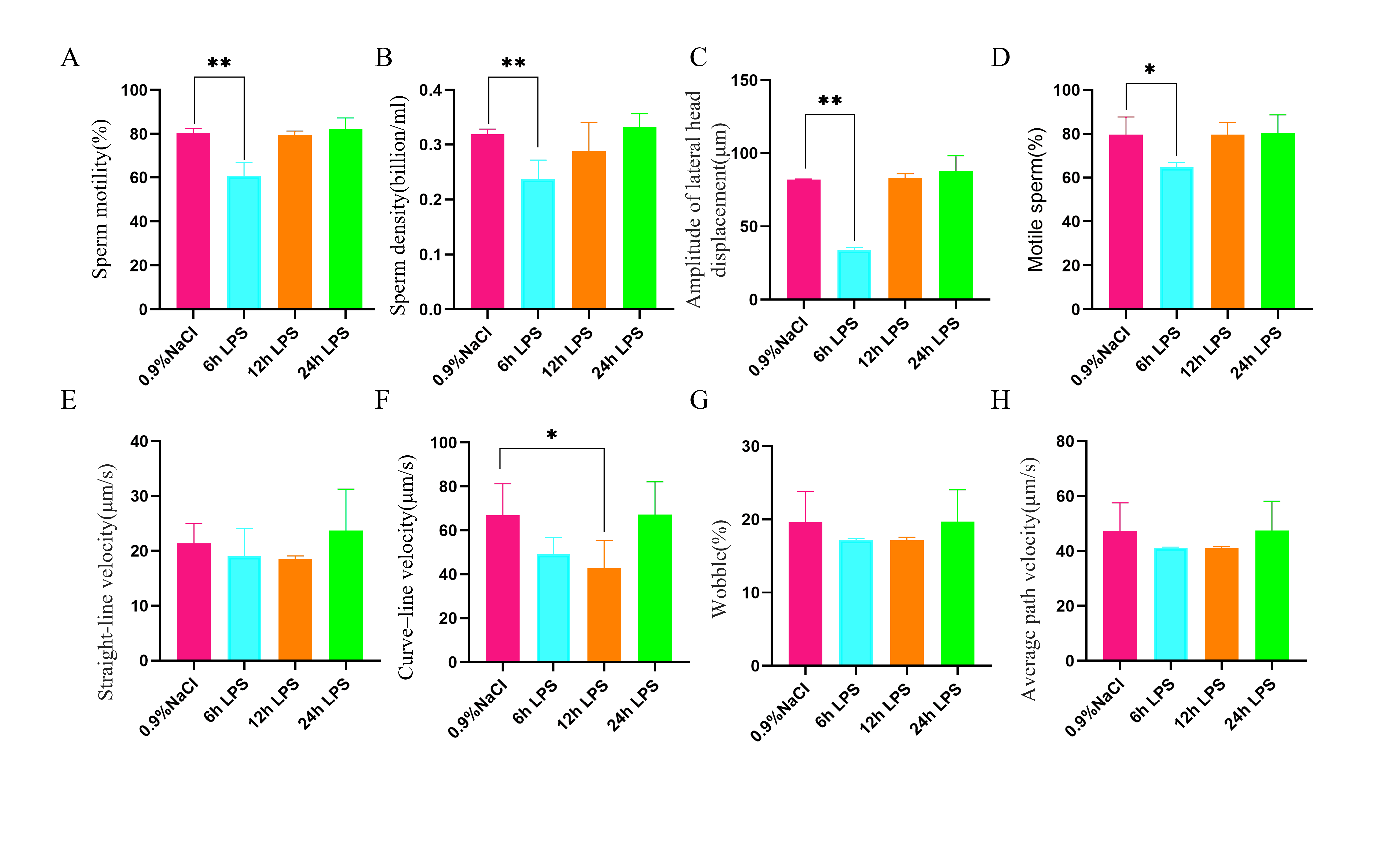

Supplement: Supplementary file 4 — Supplementary Figure 4. [file 41598_2023_45620_MOESM4_ESM.tif]

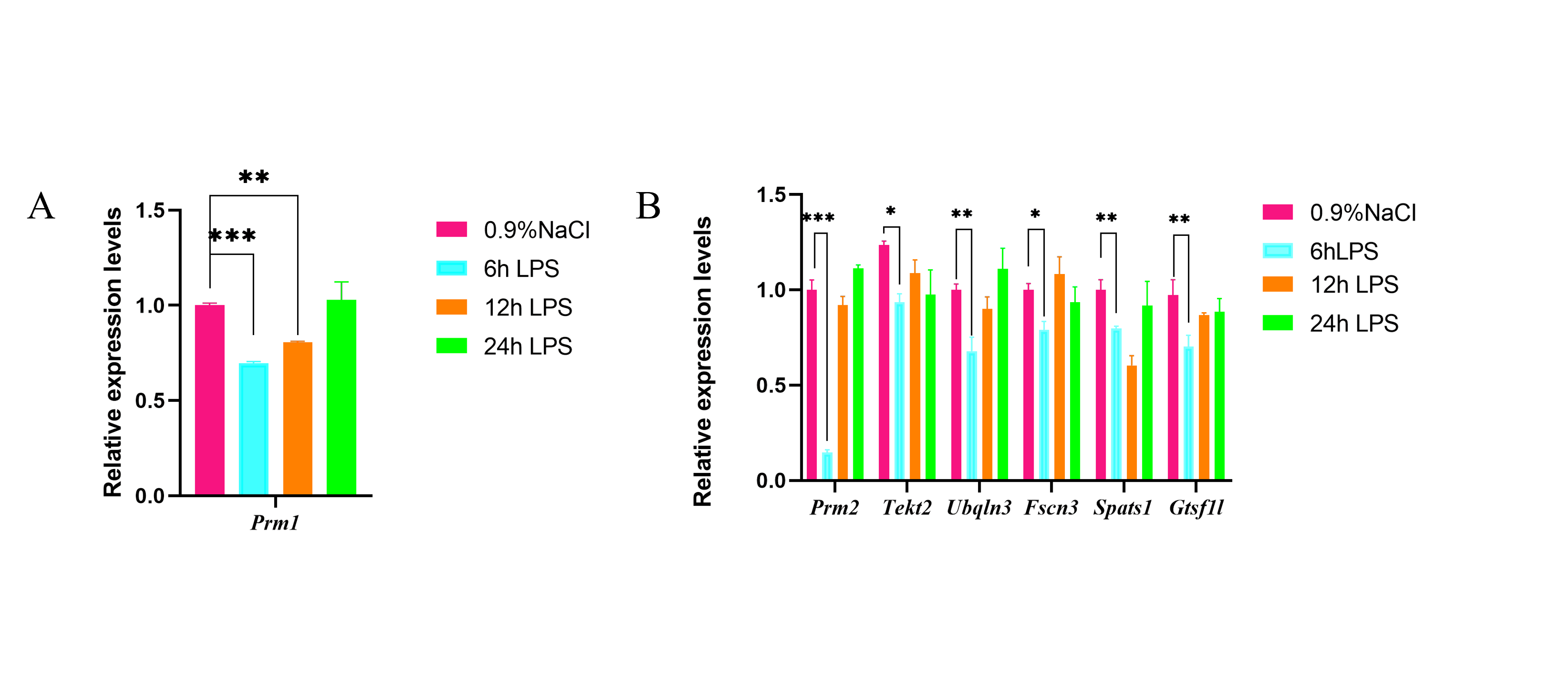

Supplement: Supplementary file 5 — Supplementary Figure 5. [file 41598_2023_45620_MOESM5_ESM.tif]
