# Supplementary material for: Identification of hub genes associated with spermatogenesis by bioinformatics analysis
Source: Sci Rep. 2023 Oct 27;13:18435. doi: 10.1038/s41598-023-45620-3 (PMC10611713; doi:10.1038/s41598-023-45620-3)
Supplement: Supplementary file 6 — Supplementary Legends. [file 41598_2023_45620_MOESM6_ESM.docx]

**Supplementary Fig. 1** Normalization and principal component analysis (PCA) of genes expressed in NOA and normal tissues. Normalization of gene expression of GSE45585 (A), GSE45587(B) and GSE9210(C). The X-axis represents the sample number, and the Y-axis represents the normalized expression value (median value). PCA of GSE45585 (D), GSE45587(E) and GSE9210(F). blue: control group; Purple: NOA group.

**Supplementary Fig. 2** A Venn diagram showing the 182 commonly expressed DEGs across GSE45585, GSE45587, GSE9210, and GSE145467 datasets. These DEGs are all part of the 203 identified by GSE45585, GSE45587, and GSE9210 datasets.

**Supplementary Fig. 3** HE staining of testis sections from acute orchitis mice at 6h, 12h and 24h after LPS injection and control group (0.9% NaCl). Scale bar; 50 µm. **P*< 0.05; ***P* < 0.01; ****P* < 0.001.

**Supplementary Fig. 4** The sperm parameters were analyzed, revealing significant decreases in sperm viability (*P*<0.01), density (*P*<0.01), amplitude of lateral head displacement (*P*<0.01), percentage of motile sperm (*P*<0.05) and curve-line velocity (*P*<0.05).

**Supplementary Fig. 5** The expression of *Prm1* and six hub genes were analyzed in the testes of acute orchitis mice induced by LPS injection. It was found that *Prm1* was down-regulated after 6 and 12 hours of LPS injection, but its expression was similar to the control group at 24 hours (A). To quantify *expression, β-actin* was used as an internal control. Additionally, all six hub genes were down-regulated after 6 hours of LPS injection in the testes of orchitis mice (B). *Gapdh* was used as an internal control for this analysis. **P*< 0.05; ***P* < 0.01; ****P* < 0.001.
